# Genome-wide SNP discovery in walnut with an AGSNP pipeline updated for SNP discovery in allogamous organisms

**DOI:** 10.1186/1471-2164-13-354

**Published:** 2012-07-31

**Authors:** Frank M You, Karin R Deal, Jirui Wang, Monica T Britton, Joseph N Fass, Dawei Lin, Abhaya M Dandekar, Charles A Leslie, Mallikarjuna Aradhya, Ming-Cheng Luo, Jan Dvorak

**Affiliations:** 1 Department of Plant Sciences, University of California, Davis, CA 95616, USA; 2 Cereal Research Centre, Agriculture and Agri-Food Canada, Winnipeg, MB R3T 2M9, Canada; 3 Genome Center Bioinformatics Core Facility, University of California, Davis, CA, 95616, USA; 4 Germplasm Repository, USDA-ARS, Davis, CA, 95616, USA

**Keywords:** BAC, Physical map, BAC end sequence, Infinium, Single nucleotide polymorphism, Genome sequence, SOLiD, Walnut, AGSNP

## Abstract

**Background:**

A genome-wide set of single nucleotide polymorphisms (SNPs) is a valuable resource in genetic research and breeding and is usually developed by re-sequencing a genome. If a genome sequence is not available, an alternative strategy must be used. We previously reported the development of a pipeline (AGSNP) for genome-wide SNP discovery in coding sequences and other single-copy DNA without a complete genome sequence in self-pollinating (autogamous) plants. Here we updated this pipeline for SNP discovery in outcrossing (allogamous) species and demonstrated its efficacy in SNP discovery in walnut (*Juglans regia* L.).

**Results:**

The first step in the original implementation of the AGSNP pipeline was the construction of a reference sequence and the identification of single-copy sequences in it. To identify single-copy sequences, multiple genome equivalents of short SOLiD reads of another individual were mapped to shallow genome coverage of long Sanger or Roche 454 reads making up the reference sequence. The relative depth of SOLiD reads was used to filter out repeated sequences from single-copy sequences in the reference sequence. The second step was a search for SNPs between SOLiD reads and the reference sequence. Polymorphism within the mapped SOLiD reads would have precluded SNP discovery; hence both individuals had to be homozygous. The AGSNP pipeline was updated here for using SOLiD or other type of short reads of a heterozygous individual for these two principal steps. A total of 32.6X walnut genome equivalents of SOLiD reads of vegetatively propagated walnut scion cultivar ‘Chandler’ were mapped to 48,661 ‘Chandler’ bacterial artificial chromosome (BAC) end sequences (BESs) produced by Sanger sequencing during the construction of a walnut physical map. A total of 22,799 putative SNPs were initially identified. A total of 6,000 Infinium II type SNPs evenly distributed along the walnut physical map were selected for the construction of an Infinium BeadChip, which was used to genotype a walnut mapping population having ‘Chandler’ as one of the parents. Genotyping results were used to adjust the filtering parameters of the updated AGSNP pipeline. With the adjusted filtering criteria, 69.6% of SNPs discovered with the updated pipeline were real and could be mapped on the walnut genetic map. A total of 13,439 SNPs were discovered by BES re-sequencing. BESs harboring SNPs were in 677 FPC contigs covering 98% of the physical map of the walnut genome.

**Conclusion:**

The updated AGSNP pipeline is a versatile SNP discovery tool for a high-throughput, genome-wide SNP discovery in both autogamous and allogamous species. With this pipeline, a large set of SNPs were identified in a single walnut cultivar.

## Background

Walnut (*Juglans regia* L., 2*n* = 32, ~606 Mb per 1C genome, Horjales et al. 2003 in http://data.kew.org/cvalues/) is an economically important tree widely cultivated for its nuts and timber. A long reproductive cycle [1] limits its genetic improvement. Walnut breeding would therefore greatly benefit from the development of molecular markers that could be used for gene discovery, marker-assisted selection, and other breeding applications that would accelerate breeding progress.

Walnut genetic markers are currently inadequate to satisfy these needs [1]. Only a limited number of amplified polymorphic DNA (RAPD) markers, RAPD-derived sequence characterized amplified regions (SCAR), restriction fragment length polymorphisms (RFLP), and amplified fragment length polymorphisms (AFLP) have been developed [2-4]. Only a few simple sequence repeats (SSR) have been developed in walnut [5-9], although the recently reported bacterial artificial chromosome (BAC) end sequences (BESs) provide an opportunity for the development of a larger number of them [10].

Single nucleotide polymorphism (SNP) is the most abundant type of DNA variation in most species. The advent of massively parallel next generation sequencing (NGS), coupled with high throughput genotyping technology, makes it relatively easy to identify and use SNPs [11-15]. An example of a high-throughput SNP genotyping platform is Illumina’s Infinium SNP oligonucleotide assay, which can simultaneously assay between 3,000 and 1 million SNPs [16,17]. The assay has been deployed in high-throughput SNP genotyping of animals, such as cattle (50 K BeadChip) [18] and swine (60 K BeadChip) [19]. A prerequisite for the development of an Infinium SNP assay is the availability of a large number of genome-wide SNPs.

Genome-wide SNP discovery utilizing NGS is predicated on bioinformatic tools facilitating mapping NGS reads to reference sequences [20-22] and variant calling [23]. Pipelines for processing of billions of short NGS reads for the purpose of discovery of genome-wide SNPs have been reported [24,25]. Standard approaches to genome-wide SNP discovery are searches for variants in transcriptome assemblies of multiple individuals or mapping of NGS genomic reads of multiple individuals to a complete genome sequence. This approach is limited in many species by the absence of a complete genome sequence, and alternative strategies for genome-wide SNP discovery are therefore needed.

One such strategy is to substitute shallow genome coverage of long sequence reads, or their assemblies, generated with the Roche 454 or Sanger sequencing technology for a complete genome sequence. This strategy was implemented in the AGSNP pipeline for genome-wide SNP discovery in self-pollinating (autogamous) plants without a reference genome sequence. In AGSNP, deep genome coverage of NGS reads from one homozygous individual was mapped to shallow 454 reads of another homozygous individual. SNPs were discovered between the two sets of reads. The assumption of homozygosity limits the universal utility of the pipeline because many plants and most animals are allogamous and hence heterozygous. We report here an update of AGSNP for applications in allogamous species.

BAC end sequences are one of several possible sources of shallow coverage, genome-wide, long DNA reads. BESs are often developed from BAC libraries and are used for marker development and genome sequence composition surveys before whole genome sequencing [10]. BESs can also be used for anchoring FPC contigs on a genetic map by searching for homology between BESs and marker sequences on the genetic map. The BES-based anchoring strategy is an alternative to contig anchoring via hybridization of radioactive probes with BAC library screening membranes [26] or screening of multidimensional BAC pools by PCR or Illumina’s Golden Gate [27-29]. The deployment of BESs in SNP discovery can therefore serve multiple objectives.

To develop markers for the construction of walnut genetic map and anchoring walnut FPC contigs on it, a total of 54,912 BESs from walnut cv ‘Chandler’, a major walnut scion cultivar grown in California, have been generated by Sanger sequencing [10]. SNPs were identified in BESs with the updated AGSNP pipeline by mapping deep sequence coverage of walnut NGS reads to ‘Chandler’ BESs. An Infinium assay for 6,000 SNPs was developed and used to genotype a ‘Chandler’ x ‘Idaho’ F_1_ mapping population. The genotyping results were analysed to validate the SNPs and to improve the SNP discovery rate with the pipeline by adjusting the SNP filtering criteria.

## Results

### Updating AGSNP

**Figure 1:**
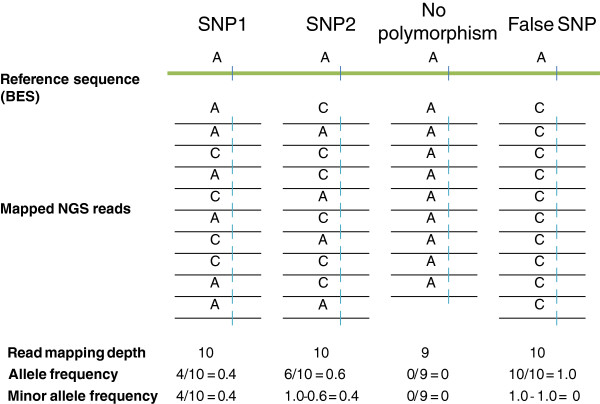
**SNP discovery strategy with a reference sequence and short NGS reads of a heterozygous diploid genome.** Variant frequency (*VF*) is defined as the ratio of the number of mapped NGS reads within a stack with a nucleotide different from the nucleotide in the reference sequences divided by total number of mapped reads in the stack. The domain of *VF* is [0,1]. Folded variant frequency (*FVF*) equals to 1-*VF* if *VF*> 0.5 and *FV* if *VF* ≤ 0.5. The domain of *FVF* is [0.0, 0.5]. SNP1 and SNP2 have different *VF* values but the same *FVF*. Cutoff values for *VF* or *FVF* and read mapping depth must be optimized to reduce the false-positive SNP rate resulting from sequencing and mapping errors. The SNP3 is inferred to be a true SNP and the nucleotide in the reference sequence is inferred to be a sequencing error.

Walnut is an outcrossing species, and walnut cultivars, such as ‘Chandler’, are highly heterozygous clones propagated by grafting. NGS reads generated from a single walnut cultivar can consequently be derived either from a single haplotype if a locus is homozygous or from two different haplotypes if the locus is heterozygous. Hence, observing two different nucleotides at a nucleotide position in a stack of mapped reads should be used as evidence for an SNP (Figure 1). Because the original version of AGSNP was designed for mapping NGS reads of a homozygous line, observing two different nucleotides at a nucleotide position was used by AGSNP as evidence for the presence of a paralogous sequence or a sequencing error and was filtered out. To update AGSNP for SNP discovery in heterozygous individuals, such as those of walnut, a new script was added to the pipeline to handle SNP discovery using reads derived from potentially heterozygous loci. The updated pipeline program is available at http://avena.pw.usda.gov/wheatD/agsnp.shtml.

Updating the AGSNP pipeline was based on the following rationale. Reads forming a stack may vary either due to heterozygosity or sequencing and mapping errors. If heterozygosity was the cause of variation, two variants were expected at a nucleotide position in a stack of reads, and the expected frequency of each variant was 0.5. If a sequencing or mapping error was the cause of variation, two or more variants were expected at a nucleotide position in a stack of reads, and the frequency of one of the variants was expected to be minor. Variables ‘variant frequency’ (*VF*), defined as the number of SOLiD reads in a stack having a nucleotide at a specific nucleotide position that was different from the reference sequence divided by the total number of reads in the stack and ‘folded variant frequency’ (*FVF*), derived from *VF* were used to discriminate between these two possibilities. *FVF* is 1-*VF* if *VF > 0.5* and equal to *VF* if *VF ≤ 0.5*. The domain of *VF* was 0 to 1 and that of *FVF* was 0 to 0.5. If *FVF* was 0, the locus was homozygous. If *FVF* was minor, read variation was likely a sequencing or mapping error and if it was near 0.5, read variation was likely caused by SNP.

**Figure 2:**
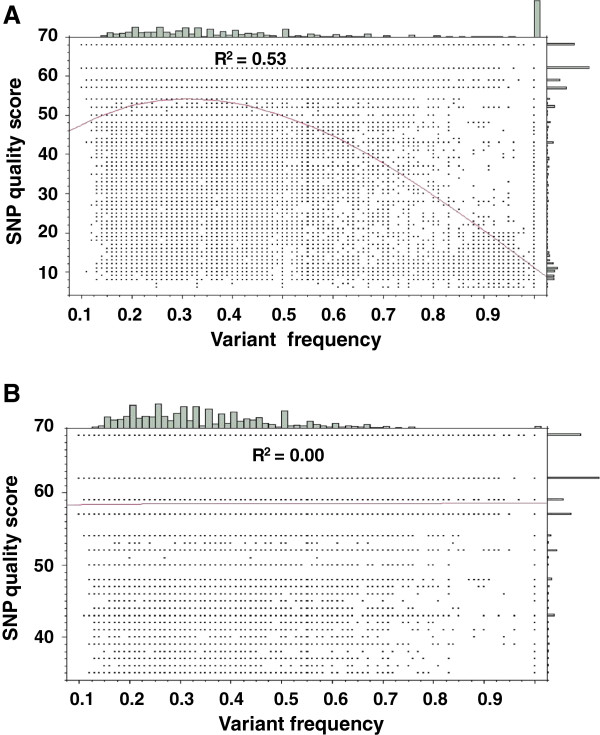
**Relationship of reference sequence quality scores of putative SNPs and variant frequency (*VF*).** High correlation between reference sequence quality scores of putative SNPs and *VF* was observed in the data set of all putative SNPs (**A**). When putative SNPs with a Sanger quality score of less than 35 were removed, the correlation disappeared, showing that SNPs with high *VF* are false-positive SNPs due to sequencing errors in BES Sanger sequences (**B**).

It was observed that the distribution of *VF* was skewed towards small values (Figure 2) and that *FVF* was significantly correlated to the quality scores of Sanger BES sequences (Figure 2 and Table 1). Both observations were consistent with the assumption that sequencing errors have low *FVF.* We therefore set *FVF* < 0.2 as a cut of for filtering out false SNPs. The final *FVF* cut off value was based on a statistical test (see Methods). If the test indicated that *FVF* at a nucleotide position was not significantly different from 0.5, the variant nucleotide was assumed to be a true SNP. A special case was when *FVF* > 0 but the stack of reads varied for two nucleotides, both differing from the reference sequence (Figure 1, SNP3). If *FVF* did not statistically differ from 0.5, variation of the reads was assumed to be caused by an SNP and the nucleotide in the reference sequence was assumed to be a BES error.

**Table 1:** **Numbers of putative SNPs in relation to read variant frequency (*VF*)**

***VF***	**No of all SNPs**	**No of SNPs after removing SNPs with quality score < 35**
1.0	13,562	255
≥ 0.9	15,261	340
≥ 0.8	17,434	593
≥ 0.7	19,425	1,448
≥ 0.6	23,330	3,959
≥ 0.5	30,441	9,602
≥ 0.4	40,495	17,863
≥ 0.3	56,195	30,687
≥ 0.2	74,744	45,976
≥ 0.1	81,986	52,006

### Discovery and characterization of SNPs

A total of 48,661 BESs of an average read length of 721 bp and totaling about 35 Mbp were used as reference sequences. Of them, 42,022 (86%) were located in 804 of the 916 FPC contigs assembled from fingerprinted BAC clones (not shown), which indicated that the BESs were distributed across the entire walnut genome. To annotate these BESs, homology was searched between the 48,661 BESs and walnut cDNA sequence contigs at 1E-10. A total of 29,223 BESs showed homology to cDNA sequences. Those BES were called genic BESs whereas the remaining 19,438 were called non-genic BESs.

A total of 395,528,231 high-quality SOLiD reads 50 bp long were retained after removing low quality reads with an average read quality score < 20. The total length of the reads was 19,776 Mbp, which translated to ~32.6X walnut genome equivalents. The filtered SOLiD reads were mapped to the 48,661 reference BESs using the BWA program [20,21], and SNPs were called in the mapped SOLiD reads using SAMtools [23].

The SNP filtering criteria initially used are summarized in Table 2. A total of 14,829 putative SNPs from 9,672 genic BESs and 7,970 SNPs from 5,401 non-genic BESs were identified (Table 3). They were present in approximately 31% of BESs and their frequency was one SNP per 1,498 bp. Genic BESs had higher SNP frequency (one SNP per 1,383 bp) than non-genic BESs (one SNP per 1,712 bp).

**Table 2:** SNP filtering criteria used in walnut SNP discovery

	**Item**	**Initial criteria**	**Adjusted**
1	Minimum read depth mapped to the reference sequences (Minimum *RMD*)	≥ 5	
	Maximum read depth mapped to the reference sequences (maximum *RMD*)	No constraint	≤ 25 ( $\overset{￣}{X}$+ 0.5- *s)*^(a)^
2	Folded variant frequency in SOLiD reads (*FVF*)	≥ 0.2	Statistically no deviation from 0.5^(b)^
3	Mapping quality score in SAMtools (*MQS*)	≥ 25	≥ 30
	Reference SNP base quality	SNP base ≥40 for genic BESs and ≥45 for non-genic BESs	
4	Removing homopolymer SNPs	SNP base string length ≥ 3 bp	
5	Removing very close SNPs	> 3 bp between two contiguous SNPs	
6	Removing SNPs at the right side of Sanger reads	> 30 bp away from the right side	
7	Illumina genotyping quality	≥ 60 bp between two contiguous SNPs	

(a) See the text for definition of $\bar{X}$ and *s*. (b) See Methods.

**Table 3:** Putative SNPs identified from the walnut BESs and their Infinium types and ADT design scores for Infinium genotyping

**BES**	**No of BESs**	**No of BESs with SNPs**	**Total SNPs**	**Infinium I SNPs**	**Infinium II SNPs**	**Infinium II SNP Design score ≥ 0.9 (%)**	**Infinium II SNP Design score ≥ 0.7 (%)**
Genic	29,223	9,672	14,829	1,810	13,019	11,112 (85.4%)	12,891 (99.0%)
Non-genic	19,438	5,401	7,970	897	7,073	5,997 (84.8%)	7,000 (99.0%)
Total	48,661	15,073	22,799	2,707	20,092	17,109 (85.2%)	19,891 (99.0%)

SNPs are divided into two categories in the Infinium HD assay, Infinium I type (A/T, C/G) and Infinium II type (A/C, A/G, T/C, T/G), according to probe or bead type design. The Infinium II probe design employs one probe per SNP (single probe for both alleles) whereas Infinium I probe design employs two probes (one probe for each allele). As the pricing and ordering of the custom BeadChip product are determined by the number of bead types, rather than the number of SNPs, using only Infinium II SNPs increases the number of SNP loci genotyped per constant number of probes and is therefore more economical. In this study, 88% (20,092/22,799) of SNPs were of Infinium II type (Table 3). SNPs of Infinium II type were present in 11,247 BAC clones present in 683 FPC contigs containing 107,262 BAC clones, which accounted for 94.9% of the walnut physical map.

### SNP genotyping

All of the 22,799 putative SNPs identified in genic and non-genic BESs were evaluated using Illumina’s ADT software. In the 20,092 SNPs of Infinium II type, 17,019 SNPs (85.2%) had a score ≥0.9, and 19,891 (99%) of SNPs had a score ≥ 0.7 (Table 3). The design score of 0.7 was used as a cutoff. After removing SNPs with design score < 0.7, 16,216 SNPs located in 682 FPC contigs were retained. A total of 6,000 of them, 3,866 from genic BESs and 2,134 from non-genic BESs, were chosen for designing a 6 K Infinium SNP assay and genotyping of 352 F1 walnut plants making up the mapping population from the ‘Chandler’ x ‘Idaho’ cross.

**Figure 3:**
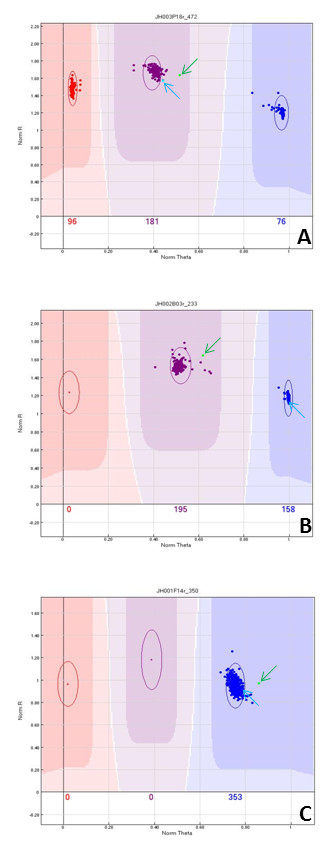
**Genome Studio outputs of Manhattan distance graphs of three different SNPs assayed with the 6 K walnut SNP Infinium assay. A** shows both parents, ‘Chandler’ (green dot indicated by green arrow) and ‘Idaho’ (light blue dot indicated by blue arrow), heterozygous at a locus. F_1_ progeny segregation (96:181:76) approximates the 1:2:1 monohybrid segregation ratio of two codominant alleles. **B** shows ‘Chandler’ heterozygous and ‘Idaho’ homozygous at a locus. F_1_ progeny segregation (195:158) approximates the 1:1 monohybrid back-cross segregation ratio. **C** shows both parents monomorphic at the locus and no segregation (353:0) of F_1_ progeny.

Visualization of genotyping data with the Genome Studio program showed that 5,420 of the 6,000 SNP markers produced genotyping data (Additional file 1). Based on the genotypes of cv ‘Chandler’ and ‘Idaho’ and their F1 progeny, 5,163 SNP markers generated good clustering in the Genome Studio graphs. Of these, 880 SNP markers generated 1:2:1 clustering of the mapping population, like an F_2_ (Figure 3A), 1,695 SNP makers generated 1:1 clustering, like a test-cross (Figure 3B), and 2,588 SNP markers generated a single cluster indicating no polymorphism between the parents (Figure 3C). Of the 5,420 SNP markers, 257 (4.7%) did not cluster accurately enough for genetic mapping although they could be scored. The conversion rate from discovered SNPs to potential SNP markers was 86.1% (5,163/6,000). Hence, 2,318 of the 5,420 functional assays (42.7%) that made up the 6 K Infinium generated data that could be used for the intended purpose, the construction of a walnut genetic map and anchoring of FPC contigs on it.

**Figure 4:**
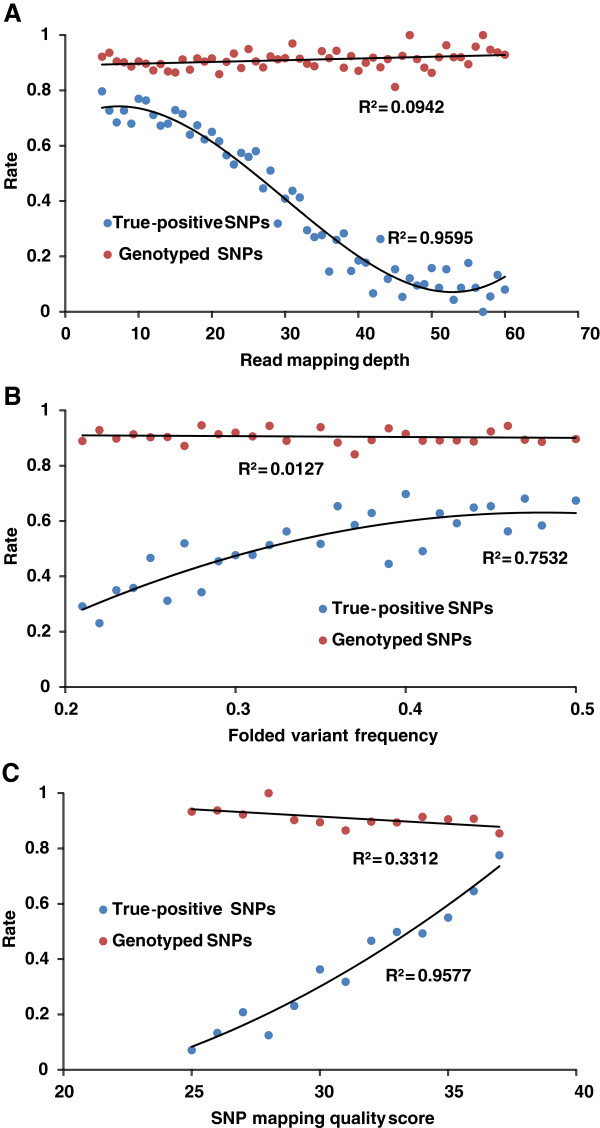
**Relationship of read (*RMD*) (A), folded variant frequency (*FVF*) (B), and SNP mapping quality score (*MQS*) (C), with the rate of true** - **positive SNPs (blue dots) and the rate of genotype SNPS.** Graphs are based on Infinium genotyping data of 6,000 selected SNPs and corresponding data of *RMD*, *FVF* and *MQS* in SNP discovery. The rate of true-positive SNPs was the number of true SNPs at an *RMD*, an *FVF* or an *MQS* value divided by the total number of genotyped SNPs (including both true- and false-positive SNPs) at the specific *RMD*, *FVF* or *MQS*. Similarly, the rate of genotyped SNPs was the number of genotyped SNPs at a specific *RMD*, *FVF* or *MQS* divided by the total number of SNPs (including both failed and genotyped SNPs) at the specific *RMD*, *FVF* or *MQS*.

To examine the reasons why 580 SNP markers failed to generate scorable genotyping data, ADT design score and the following five SNP discovery criteria or variables were compared between the 5,420 genotyped SNPs and 580 unscorable SNPs. The criteria were: average reference quality score (based on average BES quality score), reference SNP base quality score, SNP mapping quality score (*MQS*) (from the BWA mapping software [20]), *RMD*, and *FVF* (Table 4). No significant difference was observed between the two groups in 5 of the 6 examined variables. An exception was *RMD* (*p* = 0.0086) (Table 5). However, further analysis revealed no significant correlation between the rates of genotyped SNPs and *RMDs* (*R*^*2*^ = 0.0942) (Figure 4A). Among the 580 unscorable SNPs, 378 were derived from genic BESs and 202 from non-genic BESs, while the remaining 5,420 genotyped SNPs included 3,488 SNPs from genic BESs and 1,932 SNPs from non-genic BESs. A contingency *χ*^2^ test indicated that there was no significant association between SNP source and SNP genotyping success (Fisher’s exact test, *p* = 0.71). It was therefore concluded that unscorable SNPs were most likely due to random effects in the Infinium genotyping assay itself while ADT design score (≥ 0.7) and other SNP discovery factors did not impact the success rate of the conversion of SNPs to markers.

**Table 4:** Comparison of several SNP identification criteria and ADT design scores in scorable and unscorable SNPs

**SNP**	**Number of SNPs**	**ADT design score**	**Average quality score of reference**	**SNP quality score of reference**	**SNP mapping quality score**	**Read mapping depth**	**Folded variant frequency**
Scorable	5,420	0.973 ± 0.052^(a)^	54.6 ± 9.8	59.1 ± 6.8	34.0 ± 1.8	24.9 ± 15.7	0.35 ± 0.10
Unscorable	580	0.977 ± 0.047	54.5 ± 9.5	59.2 ± 6.9	34.0 ± 1.8	23.1 ± 14.1	0.35 ± 0.10
*P* value of *t* test		0.0677	0.9177	0.7894	0.8213	0.0086**	0.9896

(a) average ± standard deviation; ** represents statistical significance at a 0.01 probability level.

**Table 5:** Comparison of several SNP filtering criteria and ADT design scores in true-positive and false-positive SNPs

**SNP**	**No of SNPs**	**ADT design score**	**Average quality score of reference**	**SNP quality score of reference**	**SNP mapping quality score**	**Read mapping depth**	**Folded variant frequency**
True-positive	2,765	0.974 ± 0.050^(a)^	54.9 ± 9.4	59.2 ± 6.6	34.3 ± 1.6	18.4 ± 9.3	0.39 ± 0.11
False-positive	2,655	0.971 ± 0.053	54.3 ± 10.1	59.1 ± 7.1	33.7 ± 2.0	31.7 ± 18.0	0.34 ± 0.11
*P* value of *t* test		0.0597	0.0136*	0.4553	<0.0001**	<0.0001**	<0.0001**

(a) average ± standard deviation; * and ** represent statistical significance at 0.05 and 0.01 probability level, respectively.

### Optimization of the pipeline

**Figure 5:**
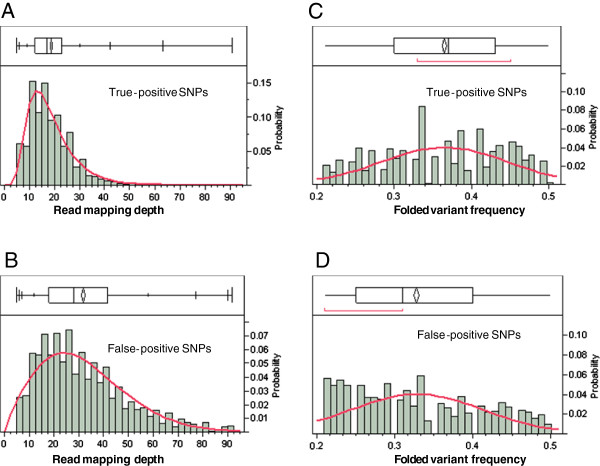
**Frequency distribution (bottom of each figure) and quantile box (top of each figure) of SOLiD read mapping depths in walnut BESs (A and B) and SNP folded variant frequency (*FVF*) (C and D) for true-positive SNPs (A and C) and false positive SNPs (B and D).** Frequencies and quantiles were computed from Infinium genotyping of 6,000 SNP markers and corresponding data of read mapping depth and *FVF* in SNP discovery.

A total of 2,588 false-positive SNPs were among 5,420 putative SNPs that could be genotyped with the 6 K Infinium assay, as evidenced by the lack of segregation in the ‘Chandler’ x ‘Idaho’ mapping population (Table 5). An additional 67 SNPs that clustered like a test-cross were inferred to be false-positive as those loci were homozygous in ‘Chandler’ (Table 5). Hence, the error rate in SNP discovery was 49.0% (2,655 false positive SNPs of 5,420 SNPs) using the initial SNP filtering criteria (Table 2). Comparison between true-positive SNPs and false-positive SNPs showed statistically significant effects in *RMD*, *FVF*, *MQS*, and average reference quality score, especially in the first three variables (Table 5). The distribution of *RMD* and *FVF* differed between true-positive and false-positive SNP groups (Figure 5). *RMD* had a sharp distribution centered at 18.4 reads in the true-positive SNP group but a flat distribution centered at 31.7 reads in the false-positive SNP group (Figures 5A and 5B and Table 5). *FVF* was closer to 0.5 in the true-positive SNP group than in the false-positive SNP group (Figures 5C and 5D).

Out of the 2,655 false-positive SNPs, 1,850 SNPs were from genic BESs and 805 SNPs were from non-genic BESs, while of the 2,765 true-positive SNPs, 1,638 SNPs were from genic BESs and 1,127 SNPs were from non-genic BESs. There was a highly significant relationship between SNP source and true/false-positive SNP outcome (Fisher’s exact test, *p* < 0.0001); SNPs from non-genic single-copy BESs had a higher chance of being true-positive (58.3%) than those from genic BESs (47.0%).

Correlation analyses revealed that the rate of true-positive SNPs (*RTP*) was significantly positively correlated to *FVF* and *MQS*, and negatively to *RMD* (Figure 4). A logistic regression model of *RTP* with *MQS* (*X1*), *RMD* (*X2*) and *FVF*(*X3*) was fitted (Table 6): log (*p*/(1-*p*)) = 10.3976899 - 0.3077982*X1* + 0.07896269*X2* - 5.2585503*X3*, where *p* is the probability of an SNP to be declared false-positive. This model was statistically significant (whole model test with *χ*^2^ = 1,592.4 and *p* = 0 and lack of fit test with *χ*^2^ = 2,027.9, *p* = 0.4905, not significant) and can therefore be used to predict whether an SNP is true-positive or not. A total of 1,777 of 2,655 false-positive SNPs were correctly predicted with this model with an accuracy rate of 66.9% and 2,203 of 2,765 true-positive SNPs were correctly predicted with this model with an accuracy rate of 79.7%, showing that the model had higher prediction accuracy for true-positive SNPs than for false-positive SNPs. The overall prediction accuracy was 73.4% (3,980/5,420). The predicted rate of true-positive genic SNPs was 53.3% (7,902/14,829) and 63.9% (5,090/7,970) of true positive non-genic SNPs. The overall rate of true-positive SNPs was 57.0%, higher than the actual rate in 6,000 SNPs (51.0%).

**Table 6:** Logistic regression model of polymorphism of SNPs (true-positive and false-positive) and several related SNP filtering criteria: log (*p*/(1-*p*)) = b0 + b1*X1 + b2*X2 + b3*X3, where *p *is the probability of an SNP belonging to a false-positive

**Term**	**Estimates of coefficients**	**Std error**	***χ***^**2**^	**Prob >** ***χ***^**2**^
Intercept	10.3976899 (b0)	0.7025	219.05	<0.0001***
*MQS* (*X1*)	−0.3077982 (b1)	0.0197	244.83	<0.0001***
*RMD* (*X2*)	0.07896269 (b2)	0.003	699.67	<0.0001***
*FVF* (*X3*)	−5.2585503 (b3)	0.3871	184.57	<0.0001***

*MQS*: SNP mapping quality score; *RMD*: read mapping depth; *FVF*: folded variant frequency. *** represents statistical significance at a 0.001 probability level.

**Figure 6:**
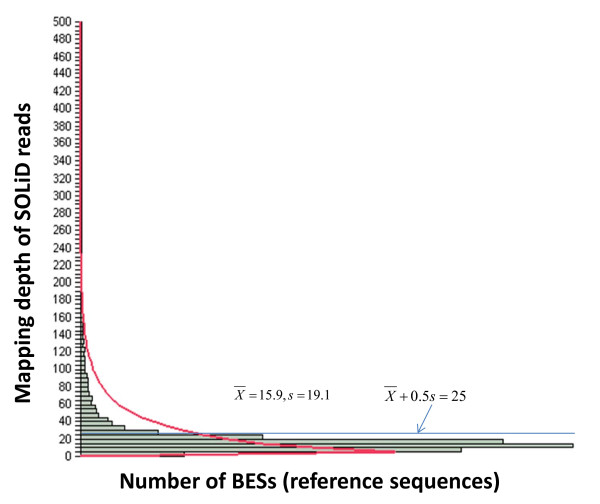
**Distribution of read mapping depths of SOLiD reads in genome equivalents to 29,223 genic BESs.** An extreme value distribution was fitted with estimates of mean ( $\bar{X}$) and standard deviation (s), 15.9 reads and 19.1 reads, respectively. The cutoff boundary after optimization of the pipeline $\bar{X}$ + 0.5s is shown.

The above analyses showed that the rate of true-positive SNPs was strongly affected by *RMD*, *MQS* and *FVF*. In order to increase the rate of true-positive SNPs generated by the pipeline in SNP discovery, the cutoff values for those three variables were modified compared to those used initially. *RMD* and *MQS* were set to 25 and 30 (Table 2), respectively. *RMD* was set to 25 based on $\bar{X}$+0.5 - *s* = 15.9 + 0.5 × 19.1 = 25 (Figure 6), which is approximately equivalent to the value of $\bar{X}$+1 - *s* = 18.7 + 9.7 ≈ 28 based on the distribution of the *RMD* among the 2,765 true-positive SNPs (Table 5 and Figure 5A). For *FVF*, a *t-*test was used to check whether the *FVF* of an SNP deviated significantly from the expected 0.5 (see Methods).

When *RMD* ≤ 25, *MQS* ≥ 30, and a non-significant difference of *FVF* from 0.5 were used to filter 5,420 SNPs used for genotyping, 69.6% (2,147/3,085) of true-positive SNPs were obtained; a much higher rate than 51.0% when initial cutoff values were used. When these criteria were used to filter all SNPs identified (Table 3), a total of 13,439 SNPs were identified in 10,313 BESs covering 677 FPC contigs, representing 98% of the physical map (Table 7). By using the logistic regression model (Table 6), 11,851 (88.2%) SNPs were predicted to be true-positive (Table 7). Considering the 73.4% prediction accuracy of the model, a true-positive SNP rate of 64.7% (0.734 × 0.882 × 100) was expected. Finally filtered SNPs and SNPs validated by the Infinium assay are in Additional files 2, 3 and 4.

**Table 7:** High-quality SNPs identified from the walnut genome after applying the new filters of read mapping depth (≤ 25), SNP mapping quality score (≥ 30), and folded variantfrequency (statistically not different from 0.5)

**BES**	**No of BESs**	**No of BESs with SNPs**	**Total SNPs**	**Number of contigs with SNPs**	**Predicted true SNP rate**^**(a)**^
Genic	29,223	6,317	8,237	629	87.1%
Non-genic	19,438	3,996	5,202	601	90.0%
Total	48,661	10,313	13,439	677	88.2%

(a) Estimated based on the logistic regression model in 2,765 validated SNPs (Table 6).

## Discussion

### Updating of the AGSNP pipeline for SNP discovery in cross-fertilizing species

The AGSNP pipeline was originally designed as a high-throughput bioinformatic tool for large-scale, genome-wide SNP discovery in large and complex genomes using sequences of two inbred, and hence homozygous, lines [25]. Sequences assembled from long reads, such as those produced by the Sanger or Roche 454 sequencing platforms, of one inbred line, and annotated using 20 cDNA libraries, served as a reference. Short reads of a great depth, such as those produced by the SOLiD or Illumina NGS platforms, of the other line were used to further annotate the assembled sequences and discover SNPs between the two lines. This pipeline was successfully used for SNP discovery in the 4.02 Gbp genome of self-pollinating *Ae. tauschii.* Approximately half of million SNPs with a validation rate of over 85.9% were identified in genic regions, single or low copy repeat regions, and uncharacterized low copy number sequences [25].

To discover SNPs in walnut, which is a wind-pollinated, out-crossing species, the pipeline had to be modified to accommodate heterozygosity. To generate SNPs for genotyping a mapping population, SNP discovery can be limited only to a single out-crossed parent. Each SNP is detected as variation within the stack of mapped short NGS reads at a locus, rather than a difference between an invariant stack of mapped NGS reads and reference sequence. In this application of AGSNP, the role of the reference sequence is to filter SNPs. Therefore, the reference sequence can be derived either from the same genotype as the mapped reads or a different genotype (Figure 1). In this study, ‘Chandler’ BESs were used as a reference sequence and SOLiD ‘Chandler’ reads were mapped to them to identify SNPs in ‘Chandler’.

SNP filtering is a critical step for removing false-positive SNPs from the pool of putative SNPs during SNP discovery. *FVF* is one of the most important variables used in SNP filtering. It is used to set a boundary between variation caused by sequencing or mapping errors and that caused by true SNPs. Ideally, *FVF* of true SNPs should be close to 0.5 but it is difficult to find a fixed *FVF* cutoff that reasonably balances false-positive and false-negative SNP rates. As the counts of variable reads at a nucleotide position follow a binomial distribution and the expected *FVF* for true SNPs is 0.5, we used the binomial probability of deviation between observed *FVF* and 0.5 to set the cutoff between true or false SNPs. The benefit of this approach to setting the cutoff value is seen using the following example. When the 5,420 genotyped SNPs were declared true or false on the basis of a fixed *FVF* cutoff of ≥ 0.3, the false-positive SNP rate insignificantly increased from 57.4% (1,525/2,655) to 61.7% (1,638/2,655). But when cutoff was set on the basis of the binomial test, the false-negative SNP rate significantly decreased from 23.1% (640/2,765) to 9.4% (260/2,765).

The SNP discovery in *Ae. tauschii*[25] used a cutoff value of $\bar{X}$+ 2 *s* to identify single copy reference sequences or to set a maximum read mapping depth. In contrast, in walnut, $\bar{X}$+ 0.5 *s* turned out to be an optimal cutoff value. Similarly, more stringent SNP filtering cutoff values were required for the SNP mapping quality score, average reference quality score, and reference SNP quality score. After adjusting the cutoff values, a 69.6% true-positive SNP rate was obtained in walnut, which was much higher than 51.0% with the initial cutoff values. This validation rate was lower than that obtained in self-pollinating species [25,30-33] but higher than that obtained using other SNP discovery strategies in outcrossing maritime pine, loblolly pine, and sugar pine, in which SNP validation rates ranged from 36.0% to 61.5% [34-36].

### Factors reducing the rate of false-positive SNPs in SNP discovery

The analysis of validated SNPs showed that factors such as read mapping depth, SNP mapping quality score, and folded variant frequency were closely related to the rate of true-positive SNPs in the updated AGSNP pipeline. All those factors were directly or indirectly associated with a fundamental issue: mismapping of NGS reads to a reference sequence. Because reference sequences and NGS reads are derived from heterozygous loci, mismapping can easily result in a large proportion of false-positive SNPs. Focusing SNP discovery on genic regions and single-copy non-genic sequences, increasing the stringency of mapping depth, increasing the SNP mapping quality score, and increasing *FVF* will decrease false-positive SNP rate. Using paired NGS reads would probably also help since it will increase the likelihood of mapping reads to their correct locations. In addition, more stringent mapping parameters in the mapping software, e.g., the number of mismatched bases and the number of gaps, should be applied if reads from heterozygous genomes are used for SNP discovery with the AGSNP pipeline.

### Infinium genotyping

A total of 6,000 SNPs scattered along most of the FPC contigs were selected to generate a 6 K iSelect Infinium BeadChip. Of them, 90.3% produced genotyping data and 86.1% were converted to potential SNP markers for genetic mapping. This conversion rate of SNP sequence to SNP markers is higher than rates in an outcrossing tree species, maritime pine, (63.6%–74.8%) [34] using the custom Golden Gate assay but lower than the conversion rates evaluated using a custom Infinium assay in animal species, such as pig (97.5%) [19] and cattle (97.6%) [18]. The final genotyping success rate is the product of a combination of the conversion rate and the true-positive SNP rate. In this study, 86.1% conversion rate and 51.0% true-positive rate yielded a final Infinium genotyping rate of 43.9%, still higher than the rate obtained in maritime pine (25.7%) [34] using the custom Golden Gate assay. Increasing true-positive SNP rate in SNP discovery will increase the final genotyping rate. Overall, genome-wide SNP discovery using BESs and short NGS sequence reads resulted in successful SNP genotyping strategy in the heterozygous walnut genome.

### General utility of identified SNPs

**Figure 7:**
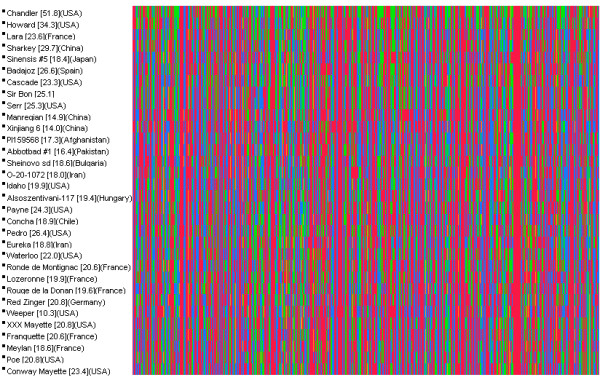
**Distribution of 5,402 SNP loci genotyped with the 6 K Infinium SNP assay in 32 walnut breeding cultivars and germplasm accessions.** The loci are arranged by their locations in FPC contigs and the location of contigs on the physical map. Bracketed values after cultivar and germplasm names are percentages of heterozygous loci. The color maps show the distribution of genotypes of loci in physical map contigs. Loci labeled blue and red are homozygous genotypes scored as AA and BB by the 6 K Infinium, respectively, and markers labeled green are the AB heterozygous loci. Note that variation is evenly distributed across the physical map in all investigated accessions. The origin of each accession is specified in parentheses.

**Figure 8:**
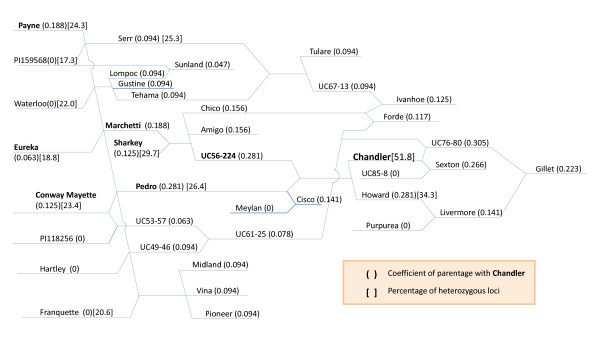
**Pedigree diagram of ‘Chandler’ and other walnut cultivars released by the UC Davis walnut breeding program.** The values in parentheses after cultivar names represent coefficients of parentage (COP) of a cultivar with ‘Chandler’. If COP is 0, the variety has no lineage relationship with ‘Chandler’. The pairwise COPs were calculated based on the definitions in [39] and [40]. The cultivars in bold font are parents or ancestors of ‘Chandler’. The percentages of heterozygous loci are in square brackets behind some cultivars.

**Figure 9:**
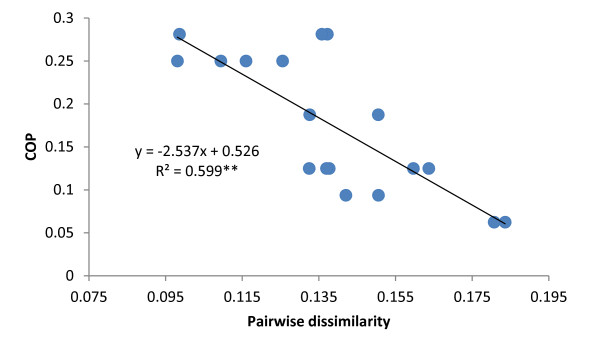
**The relationship between coefficient of parentage and dissimilarity between cultivars estimated with the walnut 6 K Infinium.** The regression equation and *r*^2^ value of the relationship are shown.

The SNPs reported here were produced for the construction of a genetic map based on the mapping population ‘Chandler’ x ‘Idaho’ and for the anchoring of FPC contigs built from the ‘Chandler’ BAC clones on the genetic map. SNPs in the ‘Chandler’ genome were therefore of a critical importance for the objectives of this project, and SNP discovery was therefore focused solely on the ‘Chandler’ genome. It is nevertheless of interest to evaluate the utility of SNPs discovered in ‘Chandler’ for other applications. To answer this question, 30 walnut cultivars, in addition to ‘Chandler’ and ‘Idaho’, were genotyped with the 6 K Infinum and their heterozygosity and pairwise genetic dissimilarity between them were estimated. As expected, heterozygosity (Figure 7) detected with the 6 K Infinium was dependent on the degree of relatedness to ‘Chandler’ (Figure 8), as indicated by positive correlation (*r* = 0.66, significant at *p* = 0.05) between heterozygosity and coefficient of parentage (COP) (Figure 8). Therefore, the diversity of walnut germplasm distantly related or unrelated to ‘Chandler’ would be underestimated if assessed by the 6 K Infinium assay, and only about a third to half as many polymorphic SNP markers would be found in germplasm distantly related or unrelated to ‘Chandler’ compared to ‘Chandler’ (Figure 7). Nevertheless, the numbers of SNPs may still be adequate and informative in many breeding and genetic applications including phylogenetic studies. That is indicated by genome-wide distribution of SNPs in all studied walnut accessions (Figure 7) and by high correlation *r* = −0.77 (significant at *p* =0.01) between pairwise dissimilarity among the 32 walnut accessions and COP (Figure 9). The agreement between the two measures of relatedness was about 60%.

## Conclusions

The AGSNP pipeline was updated here and is now applicable to genome-wide SNP discovery in all species, irrespective of their mating system, although the error rates of SNP discovery with the pipeline are higher in autogamous species (81.3 to 88%) than in allogamous species (69.6%). The greater fidelity of SNP discovery in autogamous species is undoubtedly related to greater efficiency with which the pipeline is able to separate SNPs from sequencing and mapping errors in autogamous species. The updated pipeline can be downloaded at http://avena.pw.usda.gov/wheatD/agsnp.shtml.

## Methods

### Shotgun SOLiD sequencing

A fragment library was constructed from genomic DNA isolated from the walnut cultivar ‘Chandler’ using the Applied Biosystems Fragment Library Construction Kit (Life Technologies, Inc.). Templated beads were prepared from the fragment library using the ePCR kit v.2 and the Bead Enrichment Kit from Applied Biosystems for SOLiD4+. Workflow Analysis was done after the first round of templated bead preparation for each library using the Workflow Analysis kit from Applied Biosystems to check library quality and the amount of templated beads generated per ePCR. Additional Templated beads were deposited on slides using the Bead Deposition kit from Applied Biosystems. One full slide of the fragment library was sequenced. Greater details of SOLiD library preparation and sequencing were published earlier [25].

### cDNA sequences

A total of 20 tissue-specific mRNA-Seq libraries were constructed and sequenced on the Illumina GAII platform to characterize the walnut transcriptome. Over 1 billion RNA-Seq reads were generated and trimmed for quality with a custom script. The trimmed reads derived from each sample were assembled using velvet v1.12/oases v1.15 [37] and tgicl/CAP3 [38]. Assemblies at least 200 bp long were saved and redundancy among the contigs and singletons (128,286 sequences) was removed by mapping raw Illumina RNA-Seq reads to all assembled contigs and singletons from CAP3 with BWA [20,21]. A threshold of 10 reads per kilobase mapped was set to arrive at a final set of transcriptome contigs (85,045 sequences, with a total of 137,069,830 bp and an average contig length of 1,612 bp). These sequences were used for the identification of genic BES.

### BAC contig assembly

FPC BAC contigs were constructed from 113,063 fingerprinted *Hin*dIII and *Mbo*I BAC clones of walnut cultivar Chandler [10]. A total of 917 contigs and 4,830 singletons were obtained from 108,233 clones suitable for contig assembly. Contigs can be found at ( http://probes.pw.usda.gov:8080/walnut/Database) but details of contig assembly and the construction of walnut physical map will be published elsewhere.

### BAC end sequences

The development of BESs for *Mbo*I and *Hin*dIII BAC clones has been described in detail previously [10]. A total of 54,912 BESs were produced and used here as reference sequences for SNP discovery. BES quality scores were used for SNP quality checks in the AGSNP pipeline [25].

### SNP discovery

The AGSNP pipeline [25] was updated for the discovery of SNPs in genomic sequences of a heterozygous individual. The following strategy for SNP discovery with the updated pipeline was followed. (1) Walnut BESs were annotated as genic and non-genic using blast searches against the walnut cDNA sequences. (2) SOLiD reads were mapped to the annotated BESs using the BWA program package [20] and potential SNPs were found using SAM tools [23]. (3) The maximum mapping depth cutoff value was computed according to the extreme value distribution [25] to find high-quality SNPs. (4) SNP filtering criteria were adjusted and applied to SNP discovery. The details of implementation of the updated pipeline were described in Results.

The criteria used for SNP filtering are listed in Table 2. In the previous application of AGSNP for SNP discovery in an inbred line of *Aegilops tauschii*, SNPs located in repeated sequences and those due to mapping errors were removed using the average read mapping depth (*RMD*) ( $\bar{X}$) and standard deviation (*s*) estimated from the fitted extreme value distribution of mapping depths of all mapped sequences used in SNP discovery. A cutoff value of $\bar{X}+2s$ of mapped sequences was used as a boundary between single copy reference sequences and multi-copy reference sequences [25]. Reference sequences of *RMD* less than this value were assumed to be single copy and those greater than this value were assumed to be repeated. The same strategy was used in this study. The filtered SOLiD reads were mapped to the 29,223 genic BESs using the updated AGSNP pipeline. The *RMD* ( $\bar{X}$) of SOLiD reads and standard deviation (*s*) were estimated to be 15.9 reads and 19.1 reads, respectively (Figure 4). However, as walnut is heterozygous, no data is available to suggest that a cut off of $\bar{X}$+2 - *s* is applicable as a boundary separating single copy sequences from repeated sequences in the reference sequences. To evaluate the relationship of *RMDs* with true-positive SNPs, we did not limit the maximum *RMD* in the initial SNP discovery. A more relevant *RMD* cutoff value was determined later, after SNP validation.

Folded variant frequency (*FVF*) is one of the criteria to filter out potentially false-positive SNPs among reads generated for an outcrossing individual. *FVF* represents the frequency of minor read variants in a stack of reads, which follows a binomial distribution and is expected to be 0.5 in a random mating population. A *t*-test was used to test whether or not the *FVF* of an SNP statistically deviated from 0.5 (H_0_). If the *FVF* is significantly different from 0.5 at the 0.05 probability level, the SNP was inferred to be false-positive. The test statistics is as follows:

$t=\frac{|FVF-0.5|-0.5/RMD}{0.5\sqrt{1/RMD}}$, if *RMD* × 0.5 < 30, or $t=\frac{\left|FVF-0.5\right|}{0.5\sqrt{1/RMD}}$, if *RMD* × 0.5 ≥ 30. If t ≥ t_0.05, RMD-1_, the *FVF* of an SNP is significantly different from 0.5 and thus is discarded, where t_0.05, RMD-1_ is a critical value of the *t* distribution at the 0.05 probability level with a degree of freedom of *RMD*-1.

### Infinium iSelect construction and genotyping

All SNPs identified in BESs were submitted to Illumina for evaluation using Illumina’s Assay Design Tool (ADT). A total of 6,000 SNPs were selected for iSelectInfinium genotyping. To obtain a dense, genome-wide genetic map, SNP markers should be distributed evenly across the entire genome or be present in all FPC contigs. To maximize the likelihood of that, SNP selection was based on the following criteria: (1) only one SNP was chosen per BES, (2) at least one SNP marker was chosen per FPC contig, (3) the number of selected SNPs per FPC contig was proportional to contig size, and SNPs were evenly distributed along the contig, (4) if the same gene was in multiple BESs only one BES was chosen, and (5) only SNPs of Infinium II type were used.

A mapping population consisting of 428 F_1_ progeny produced from across between cultivars ‘Chandler’ and ‘Idaho’ was used for genotyping. The F_1_ individuals, along with their parents, were grafted on to ‘Paradox’ rootstock or grown on their roots in the field. The mapping population was segregating for a number of phenological and metric traits. A set of 20 microsatellite loci were used to confirm that the individuals in the mapping population were true hybrids.

### SNP validation

The standard PCR-based approach of SNP validation by designing primers flanking an SNP, sequencing amplicons, and comparing them with expected genotype is not strictly applicable for a heterozygous genome due to possible amplification artifacts. SNPs were therefore validated indirectly through Infinium genotyping. SNPs were declared to be false positive if no polymorphism was observed at that nucleotide in cv ‘Chandler’ and its F_1_ progeny from the cross with cv ‘Idaho’.

### Pedigree analysis

Pedigrees of walnut cultivars including ‘Chandler’ released by the UC Davis walnut breeding program were collected from annual breeding progress reports and published papers [4,9]. The pairwise coefficients of parentage (COP) between cultivars in pedigrees were calculated based on the definition in Malecot [39] and Kempthorne [40]. Due to the outcrossing nature of walnut, the inbreeding coefficient (F) of a cultivar was set to 0. A Perl program ‘calculate_COP.plx’ was written for pairwise COP calculations.

### Estimation of heterozygosity and pairwise dissimilarity

In order to assess the utility of SNPs discovered in the single cultivar ‘Chandler’, a total of 32 walnut cultivars including ‘Chandler’ and ‘Idaho’ were genotyped with the 6 K Infinium genotyping assay. The heterozygosity percentage for each was calculated as the number of heterozygous loci divided by the total number of SNP markers. The pairwise dissimilarity coefficients were computed based on heterozygosity data using the improved coefficient definition and calculation methods for diploids and codominant markers [41].

### Statistical analysis

All statistical analyses, including significance test, correlation analysis and logistic regression modeling, were performed using JMP 7.0 (SAS Institute Inc.) and Microsoft Excel (Microsoft).

## Abbreviations

SNP: Single nucleotide polymorphism; BAC: Bacterial artificial chromosome; BES: BAC end sequence; RAPD: Random amplified polymorphic DNA; SCAR: Sequence characterized amplified region; AFLP: Amplified fragment length polymorphism; SSR: Simple sequence repeats; NGS: Next generation sequencing; PCR: Polymerase chain reaction; FPC: Fingerprinted contig; *RMD*: Read mapping depth; *VF*: Variantfrequency; *FVF*: Folded variant frequency; ADT: Assay design tool; *MQS*: Mapping quality score; *RTP*: Rate of true-positive SNPs.

## Competing interests

The authors declare that they have no competing interests.

## Authors’ contributions

FMY, AMD, CAL, MA, JNL, DL, JD and MCL planned the work. KRD performed SOLiD sequencing. MTB performed cDNA sequence assembly. FMY performed SNP pipeline improvement, data analysis, SNP discovery, and SNP selection and design for the Infinium assay. MCL and JW conducted Infinium genotyping and data analysis. FMY drafted the manuscript and JD revised it. All authors read and approved the final draft of the manuscript.

## Supplementary Material

Additional file 1 List of 6,000 SNPs used in the Infinium assay and their features. Click here for file

Additional file 2 List of the 13,439 discovered SNPs. Click here for file

Additional file 3 Sequences of 6,000 SNPs used in the Infinium assay. Click here for file

Additional file 4 Sequences of discovered 13,439 SNPs. Click here for file
